# Biomarkers and recent advances in the management and therapy of sickle cell disease

**DOI:** 10.12688/f1000research.6615.1

**Published:** 2015-10-12

**Authors:** Marilyn J. Telen

**Affiliations:** 1 Duke University School of Medicine, Box 2615, Duke University Medical Center, Durham, NC, 27710, USA

**Keywords:** sickle cell, anemia, biomarkers, red blood cells

## Abstract

Although production of hemoglobin S, the genetic defect that causes sickle cell disease (SCD), directly affects only red blood cells, the manifestations of SCD are pervasive, and almost every cell type and organ system in the body can be involved. Today, the vast majority of patients with SCD who receive modern health care reach adulthood thanks to vaccine prophylaxis and improvements in supportive care, including transfusion. However, once patients reach adulthood, they commonly experience recurrent painful vaso-occlusive crises and frequently have widespread end-organ damage and severely shortened life expectancies. Over the last several decades, research has elucidated many of the mechanisms whereby abnormal red blood cells produce such ubiquitous organ damage. With these discoveries have come new ways to measure disease activity. In addition, new pharmaceutical interventions are now being developed to address what has been learned about disease mechanisms.

## Introduction and context

Although it has long been straightforward to define sickle cell disease (SCD) and its subtypes through biochemical and genetic analyses of hemoglobin and its encoding genes, understanding the pathophysiologic mechanisms leading to the disease’s protean manifestations has been more challenging. After decades of research, a great deal has been learned about the many pathways and processes affected downstream by the hemoglobin S (HbS) mutation, and, finally, new therapeutic approaches targeting these mechanisms are being developed. Nevertheless, a lack of even a basic ability to document and follow the processes leading to vaso-occlusive episodes persists. At this time, both the diagnosis of vaso-occlusion and the definition of its resolution rely exclusively on patient reports of pain.

The difficulty in obtaining objectively measurable biomarkers of disease activity and variability hampers many aspects of the management of SCD, including (1) diagnosis of acute vaso-occlusion, acute chest syndrome, and other sequelae of SCD; (2) understanding of the inciting events leading to vaso-occlusion and its complications in SCD; (3) personalized medicine through identification of particularly suitable or unsuitable candidates or events for targeted disease therapies; and (4) documentation of new drug effects and mechanisms of action.

Despite the fact that the abnormal hemoglobin that defines SCD is expressed only in erythrocytes, all blood cells as well as soluble blood elements and most other organ systems are affected by SCD. Patients with SCD have, at baseline, elevated leukocyte and platelet counts, abnormally increased leukocyte and platelet activation, abnormal activation of coagulation pathways, increased expression of multiple inflammatory markers, increased expression of soluble markers of endothelial activation and injury, and increased markers associated with a broad range of end-organ damage.

Many such “biomarkers” have been studied either in “steady-state” SCD or during acute vaso-occlusive episodes, and some have been shown to correlate with long-term survival. In addition, because vaso-occlusion may arise from a variety of inciting events (e.g., infection and physiologic stress), the mechanisms of vaso-occlusion may be variable among patients and vaso-occlusive events. Identification of biomarkers specific for processes contributing to vaso-occlusion could help determine which drugs might be most beneficial and might also elucidate the mechanism of action of new therapeutic agents. Biomarkers might also help determine when new therapeutic agents might be useful as prophylactic therapy to prevent progressive end-organ damage.

The past few decades, during which both mechanisms of vaso-occlusion and biomarkers have been identified, have also brought the dawn of targeted therapies for vaso-occlusion. Although none has yet to be proven useful or become US Food and Drug Administration (FDA)-approved since approval of hydroxyurea (HU) for the prevention of vaso-occlusion and acute chest syndrome, several promising targeted agents are in various stages of clinical trials. Many such studies are also examining biomarkers, as they are affected by both vaso-occlusion and the new potential therapeutic agents. Thus, it is reasonable to expect that progress in therapeutics and in the discovery of useful biomarkers will go hand-in-hand in the future, leading potentially to both targeted and personalized therapy for SCD.

## Pathophysiology and biomarkers

### Clinical measures and overall prognosis

Early studies of the natural history of SCD, largely in children and young adults, identified several markers of disease severity and poorer overall survival ^1, 2^. Frequency of vaso-occlusive episodes was a marker of poorer survival in patients with sickle cell anemia (homozygous for HbS) who were more than 20 years old ^2^. High rates of vaso-occlusive episodes were also associated with higher hematocrit and lower fetal hemoglobin levels in that study. A second study of largely the same patient population further revealed that acute chest syndrome, renal failure, seizures, a high baseline white cell count (>15,000 cells/mm ^2^), and low fetal hemoglobin were associated with an increased risk of early death; early mortality was again shown to be more frequent among the most symptomatic patients ^1^. Finally, a more recent analysis of the cohort enrolled as newborns in the same study showed that more pronounced reticulocytosis increased the risk of stroke and mortality during childhood ^3^.

More recent studies of factors associated with mortality in SCD have presumably reflected the era of HU therapy, and some have studied factors related to survival in different resource settings. Recent studies ^4– 6^ of adults have shown that more frequent episodes of vaso-occlusion remain a marker of increased mortality ^4, 5^, and sickle nephropathy is also a significant risk factor ^4– 6^. In addition, the presence of an elevated tricuspid regurgitant jet velocity (TRV) (≥2.5 m/sec), with or without catheterization-proven pulmonary arterial hypertension, is a very significant risk factor for accelerated mortality ^4– 8^. A history of cumulative end-organ damage and stroke was also associated with earlier mortality ^5^.

Interest in pulmonary hypertension and SCD has also spurred investigation of markers thought to be related to endothelial damage and inflammation. Of these, vascular cell adhesion molecule 1 (VCAM-1) levels have been reproducibly associated with survival ^5, 9^.

Hemoglobin F (HbF) levels are often measured clinically, and several studies have supported the hypothesis that higher HbF levels lead to less severe SCD. HbF has higher oxygen affinity than HbA or HbS, and sickle red cells containing more HbF survive longer in the circulation ^10^. Moreover, elevation of HbF is a key (though not the only) factor in the salutary effects of HU on SCD severity ^11^.

### Adhesion

Sickle red cells are abnormally adherent to many substrates, including endothelial cells ^12^, leukocytes ^13^, platelets ^14^, and extracellular matrix molecules such as laminin ^13, 15^ and thrombospondin ^16, 17^. Hebbel and colleagues found evidence decades ago that patients with more adherent cells were more likely to suffer vaso-occlusive episodes ^18^. However, while such adhesion almost certainly contributes to vaso-occlusive pathogenesis, measuring adhesion either *ex vivo* or *in vivo* remains difficult. Thus, assays of cell adhesion are often used for research purposes but have not been extensively explored as markers of disease.

Nevertheless, one measurable outcome of adhesion is the formation of circulating heterocellular aggregates that can be measured by flow cytometry, now a part of most clinical hospital laboratories. Sickle red cells, as well as leukocytes from patients with SCD, can be found in circulating aggregates involving both each other as well as platelets ^14, 19^. How such measures related to clinical status and outcomes in SCD remains to be better defined.

### Inflammation

SCD is accompanied by a broad array of inflammatory processes. At steady state, in the absence of symptomatic vaso-occlusion, patients with SCD have increased numbers of activated leukocytes ^20^, activated platelets, and formation of multicellular aggregates.

In addition, patients with SCD may have elevations of multiple inflammatory cytokines ( Table 1), both in steady state as well as during vaso-occlusive events. Although not all studies demonstrate concordant findings, among the cytokines consistently found to be both elevated at steady state and then further elevated during vaso-occlusive events are interleukin-10 (IL-10), macrophage inflammatory protein 1α (MIP-1α), placenta growth factor (PlGF), prostaglandin E _2_ (PGE _2_), and soluble CD40 ligand (sCD40L). Current investigations are focusing on how these cytokines can contribute to the pathophysiology of vaso-occlusion.

**Table 1.** Expression of cytokines, acute-phase proteins, and inflammatory molecules in sickle cell disease.

Cytokine or molecule	Expression in steady-state sickle cell disease	Expression during vaso- occlusion	References
C-reactive protein	Increased	Can be further increased	102, 103
Interferon-γ	Normal or increased	Not further increased compared with steady state	104, 105
Interleukin-1β	Normal or increased	Normal or increased	104– 107
Interleukin-2	Normal	Normal	105
Interleukin-4	Increased	Not further increased compared with steady state	105
Interleukin-6	Increased	Further increased	102, 104, 105, 107
Interleukin-8	Increased	Variably further increased compared with steady state	104, 105, 107– 109
Interleukin-10	Increased	Further increased	104, 107
Monocyte chemoattractrant protein-1 (MCP-1)	Variable		102, 104
Macrophage inflammatory protein 1α (MIP-1α)	Increased	Further increased	104
Neuropeptide substance P (SP)	Increased	Further increased	108
Pentraxin-3 (PTX3)	Normal	Increased	50
Placental growth factor (PlGF)	Increased	Further increased	110– 113
Prostaglandin E _2_ (PGE _2_)	Increased	Further increased	107, 109, 114
Soluble CD40L	Increased	Further increased	91
Thrombospondin	Increased	Further increased	111
Tumor necrosis factor-α	Normal or increased	Normal or increased	102, 104– 107
TNFSF14 (LIGHT)	Increased		115

### Coagulation

Coagulation pathways are broadly activated in patients with SCD ^21^. Thus, SCD is considered a “hypercoagulable state”, and indeed there is a higher prevalence of pregnancy-related thrombosis and pulmonary emboli in patients with SCD than in age-matched African-American controls ^22, 23^. Clinically, levels of D-dimer are often chronically elevated and increase further during vaso-occlusive events ^24, 25^. Other biomarkers of activated coagulation, such as plasma levels of prothrombin fragment 1.2 (F1.2), thrombin-antithrombin (TAT) complexes, plasmin-antiplasmin complexes, and fibrinopeptide A, are also elevated in SCD. There is at least some evidence that the degree of elevation of D-dimer levels is predictive of the frequency of vaso-occlusive episodes ^26^. Furthermore, the hypothesis that abnormal SCD red cells, and specifically those with increased phosphatidylserine (PS) exposure at their surfaces, are responsible for activation of coagulation is supported by the demonstration that the number of PS-positive sickle red cells is related to the degree of elevation of D-dimer, F1.2, and plasmin-antiplasmin complex levels ^27, 28^.

Investigators have also shown that there are elevated levels of tissue factor in the circulation in SCD ^29– 31^ and that platelets are also activated in greater numbers ^32, 33^. Blood from patients with SCD also contains increased levels of microparticles derived from multiple cell types, including red cells, platelets, leukocytes, and endothelial cells. Tissue factor-expressing microparticles appear to be derived primarily from monocytes and endothelial cells ^34^. Finally, recent evidence suggests that free plasma iron may also contribute to activated coagulation in SCD ^35^.

### Oxidant damage

Oxidant damage appears to occur at an accelerated rate in SCD, both within the red cell as well as in other tissues. Hemolysis results in the release of hemoglobin, which itself is a powerful oxidant. In addition, several investigators have reported reduced anti-oxidant compounds both within sickle red cells as well as in plasma. Plasma lipid peroxidation is higher in SCD patients than controls, and red cell content of glutathione reductase and superoxide dismutase is lower in sickle erythrocytes ^36^. Depletion of glutathione has been associated with elevated TRV, itself a biomarker for pulmonary hypertension and early mortality ^37^. Finally, some evidence suggests that reduction in expression of genes responsible for the synthesis of anti-oxidant compounds may also contribute to worsening anemia in SCD ^38^. HU has been found to reduce markers of oxidative stress in SCD ^39, 40^.

### Biomarkers and stroke

Stroke is a common and potentially devastating problem in SCD, and strokes start to occur in very young children ^41^. Once a stroke occurs, patients are at great risk for recurrent strokes, which can be largely prevented by chronic transfusion but have a high frequency of recurrence without continuing transfusion ^42^. Transcranial Doppler (TCD) measurements have been known to provide a good measurement of the risk of stroke in children with SCD since 1992, when children with abnormal TCDs were shown to be at 44 times the risk for stroke than children with normal TCDs ^43^. The Stroke Prevention Trial in Sickle Cell Anemia (STOP) showed that regular transfusion could reduce the risk of a first stroke by 92%, compared with non-transfused children with abnormal TCDs, who had about a 10% incidence of stroke annually ^44^. However, a subsequent study of children who had already undergone at least 30 months of transfusion for abnormal TCDs failed to show that such transfusion could be safely stopped ^45^. Thus, while TCDs are now standard of practice in pediatric care of SCD, in order to identify children at risk for primary strokes, they do not allow avoidance of transfusion for the relatively large number of children who would not ultimately have a stroke, despite their abnormal TCDs. Efforts to better define the at-risk population, such as through identification of genetic risk factors or other biomarkers, have not yet defined a solution to this problem.

### Biomarkers of acute chest syndrome

Acute chest syndrome (ACS) is one of the most feared complications of vaso-occlusive episodes and is highly associated with mortality. Secretory phospholipase A(2) (sPLA(2)) levels become quite elevated in about 80% of patients with ACS ^46, 47^ and often become quite elevated shortly before patients become symptomatic ^47, 48^. Thrombospondin-1 levels have also been reported to become markedly increased in ACS ^49^, as have levels of pentraxin-3 ^50^. C-reactive protein has been reported to parallel sPLA(2) in the context of vaso-occlusion and ACS ^51^. At least one study has attempted to prevent ACS with transfusion in patients with high sPLA(2) levels ^52^, but a definitive study has not been conducted.

### Biomarkers and pulmonary hypertension

Pulmonary hypertension has become recognized as a major risk factor for death in adults with HbSS and HbSβ0 thalassemia ^8^. However, the most widely available screening test for pulmonary hypertension—echocardiography—does not reliably reflect pulmonary arterial pressures, as measured by right heart catheterization. In fact, right heart catheterization may confirm pulmonary hypertension in only 10% to 25% of patients with elevated TRV ^53, 54^, giving echocardiography a positive predictive value of only 25% to 32% ^55^. Nevertheless, a TRV of over 2.5 to 3 m/sec has been an indicator of risk for mortality in several studies ^6, 7, 56^ despite its lack of reliability as an indicator of catheterization-measurable pulmonary arterial hypertension ^6^. Thus, the recent National Institutes of Health guidelines for sickle cell anemia management did not recommend for or against routine echocardiographic screening for pulmonary hypertension in asymptomatic adults with SCD ^57^. In addition, several studies have shown that elevated pro-brain natriuretic peptide (pro-BNP) is associated with a high risk of mortality, especially in combination with a TRV of at least 3 m/sec ^6^. VCAM-1, a marker of endothelial activation and damage, is also present at higher levels in SCD patients with pulmonary hypertension ^58^. VCAM-1 is also reproducibly associated with poorer survival ^5, 9^.

### Biomarkers and therapeutic approaches

Although the biomarkers discussed above do not represent the totality of biomarkers explored and found to be possibly informative in the context of SCD, they do point to the panoply of pathophysiologic mechanisms now appreciated as active in SCD. Adhesion, inflammation, coagulation, and oxidative damage are likely the most important, though not the only, contributors to the development of vaso-occlusion and organ damage in this disease. Moreover, we do not completely understand how the processes implicated by the evidence of biomarkers produce the varied physiologic events we observe. For example, SCD involves both large-vessel events (e.g., strokes) as well as occlusion of microvascular structures, which is believed to be involved in typical painful vaso-occlusive events. Nevertheless, current efforts to develop new therapies for SCD are concentrating on the processes reflected by the biomarkers discussed above. In the future, biomarkers may assist us in personalizing treatment according to the predominant mechanisms involved in a particular disease sequela or event.

## New and targeted drugs in development

Since SCD was recognized and defined in the early 20th century, survival and quality of life have until recently improved primarily because of advances in supportive care, including penicillin prophylaxis, routine immunizations, transfusion for stroke and acute chest syndrome, and hydration and narcotic therapy for vaso-occlusive episodes. This changed after the Multicenter Study of Hydroxyurea ^59^, which showed that HU reduced the frequency of both vaso-occlusive episodes and acute chest syndrome while increasing both total hemoglobin levels and hemoglobin F percentages and decreasing neutrophil counts. However, the study was not designed to, and did not, determine the mechanism whereby HU reduced vaso-occlusive and acute chest syndrome events. In this setting, the FDA approved the drug for use in adults with SCD in 1998. Subsequent studies have confirmed that the drug is cost-effective ^60^ and improves long-term survival ^61– 63^. The drug has also proven to be safe and effective in children ^64, 65^. Nevertheless, the consensus remains that the drug is underutilized in both children and adults ( http://consensus.nih.gov/2008/sicklecellstatement.htm). One recent study suggested that, outside a center dedicated to treating SCD, only about 25% of patients meeting the original study criteria actually receive HU ^66^. Moreover, the mechanisms whereby HU has its beneficial effects remain only partly understood.

Not only have new therapies for SCD not arrived since HU gained FDA approval in 1998, but truly curative therapies have been difficult to achieve ( Table 2). Hematopoietic stem cell transplantation and gene therapy offer the best chances for cure but each presents numerous challenges that have been difficult to overcome. Although there were initial successes in young pediatric patients, successful hematopoietic stem cell transplantation with good survival and tolerable graft-versus-host disease in adults proved much harder to achieve. Nevertheless, such transplants can now be confidently undertaken ^67– 69^, and many ongoing clinical trials are looking at ways to improve both engraftment and the availability of donors.

**Table 2.** New therapeutic approaches under investigation or recently investigated for sickle cell disease.

Classification and agent	Rationale/Goal	ClinicalTrials.gov registration
Hematopoietic stem cell transplantation	Replace stem cells leading to production of HbS with cells that produce either normal or anti-sickling Hb (such as HbF)	00408447, 01461837, 00977691, 01877837, 02065596, and 00152113
Gene transfer or correction	Replace or correct the HbS gene with a gene encoding either normal or anti-sickling Hb (such as HbF)	02186418, 02140554, and 02151526
Nutritional supplements		
• L-glutamine	Lower energy requirements to improve growth and strength	01179217, 00131508, 00586209, and 00125788
• Niacin	Improve blood flow	00508989
• Vitamin D	Correct chronic vitamin D deficiency common in children with SCD	01276587
• Alpha-lipoic acid and acetyl-L-carnitine	Anti-oxidant	01054768
• Arginine	Improve availability of NO	00513617
Anti-inflammatory		
• Regadenoson	Downregulate the activity of iNKT cells	01566890 and 01085201
• NKTT120	Deplete iNKT cells	01783691
• Zileuton	Reduce inflammation	01136941
• Montelukast	Reduce soluble vascular cell adhesion molecule-1 (sVCAM-1)	01960413
• IVIg	Shorten VOC	01757418
Anti-adhesive		
• Propranolol	Reduce activation of red cell adhesion receptors activated via beta adrenergic receptor signaling pathways	01077921 and 02012777
• SelG1	Block adhesion via P-selectin to prevent VOC episodes	01895361
• PF-04447943	Phosphodiesterase-9 inhibitor	02114203
• Rivipansel	Reduce adhesion and inflammation dependence on E-selectin	01119833 and 02187003
Anti-sickling		
• MP4CO	Reduce sickling by delivering CO	01356485
• SCD-101	Reduce sickling	02380079
• Sanguinate (PEG-bHb-CO)	Reduce sickling by delivering CO	01848925
• AES-103	Reduce sickling	01987908
Alter hemoglobin expression		
• Vorinostat	Increase HbF	01000155
• HU with Mg Pidolate	Increase both HbF and cell hydration	00143572
• HQK-1001	Increase HbF	01601340
• Panibostat	Increase HbF	01245179
• Decitabine	Increase HbF	01375608 and 01685515
• Pomalidomide	Increase HbF	0522547
Anti-coagulant and anti-platelet		
• Ticagrelor	Decrease vaso-occlusive episodes and pain	02214121
• Prasugrel	Decrease vaso-occlusive episodes and pain	01167023, 01794000, and 01476696
• N-acetyl cysteine	Decrease VWF levels, VWF total activity, ULVWF multimers, and VWF functions	01800526
• Aspirin	Diminish the incidence and progression of cognitive deficits as well as occurrence of overt and silent stroke	00178464
• Eptifibatide	Reduce platelet activation and release of pro-inflammatory cytokines	00834899
Other		
• Inhaled NO	Improve pulmonary hypertension	00023296
• ICA-17043	Reduce hemolysis and VOC by increased cell hydration	00040677
• Intravenous NO	Vaso-dilation	00095472
• Losartan	Improve SCD nephropathy	02373241
• 6R-BH4 (sapropterin dihydrochloride)	Improve endothelial dysfunction	00445978, 00403494, and 00532844
• Magnesium sulfate	Reduce length of stay and pain in children with acute VOC	00313963
• Varespladib (A-001)	Decrease acute chest syndrome	01522196 and 00434473
• Atorvastatin	Improve endothelial dysfunction	00072826
• Simvastatin	Improve endothelial dysfunction	00508027
• Bosentan	Improve pulmonary hypertension	00313196, 00360087, and 00310830
• Clotrimazole	Reduce hemolysis and anemia by blocking Gardos channel	00004404 and 00004492

CO, carbon monoxide; Hb, hemoglobin; HbF, hemoglobin F; HbS, hemoglobin S; HU, hydroxyurea; iNKT, invariant natural killer T; NO, nitric oxide; SCD, sickle cell disease; ULVWF, ultra-large von Willebrand factor; VOC, vaso-occlusive crisis; VWF, von Willebrand factor.

Gene therapy is an even more challenging but ultimately less toxic approach to achieving curative therapy for SCD. The general approach has been to develop methods for inserting into autologous hematopoietic stem cells either a gene encoding normal β-globin or a globin chain with anti-sickling properties, such as γ-globin (that leads to production of HbF). Another approach that has been explored involves methodologies for “correcting” the faulty β-globin gene. These approaches, however, either are still in development or are in very early clinical trials.

### Inhibitors of cell activation and adhesion

One of the most attractive therapeutic targets in SCD is cell adhesion. Although SCD severity was first linked to the degree of red cell adhesion exhibited by patients’ red cells, it is now apparent that leukocyte and platelet activation and adhesion also contribute to pathophysiology. Therefore, cell adhesion has become a primary target for the development of new therapeutic approaches. In general, such approaches may involve inhibition of cell-cell interactions generally, specific inhibition of adhesion receptors, or interference with the signaling mechanisms that lead to activation of adhesion receptors.

Non-specific inhibition of cell adhesion by poloxamer-188 was studied in a multicenter randomized phase III trial involving both children and adults to determine its effect on duration of painful vaso-occlusive episodes. Although the results were statistically significant, differences in time to resolution of painful episodes were small but were slightly greater in children ^70^. Another phase III study of this drug, using a somewhat different study design, is now under way (NCT01737814, ClinicalTrials.gov).

Another anti-adhesion therapeutic has been developed to address adhesive interactions involving primarily E-selectin. This drug (GMI-1070, now known as rivipansel) was quite successful in abrogating vaso-occlusion in sickle mice ^71^. In a subsequent phase I study of SCD patients in steady state, rivipansel was well tolerated and appeared to improve blood flow in a subset of patients. Perhaps most interesting, however, was the drug’s effect on biomarkers: the drug was associated with significant decreases in biomarkers of endothelial activation, including sE-selectin, sP-selectin, and soluble intercellular adhesion molecule-1 (sICAM). Markers of leukocyte activation and coagulation were also decreased ^72^. A phase II study of the drug in patients experiencing painful vaso-occlusive episodes showed large and consistent decreases in all measures of time to crisis resolution, although these were not statistically significant. Moreover, opiate usage was markedly and statistically significantly decreased with drug versus placebo ^73^. A phase III study is expected to be under way shortly (NCT02187003, ClinicalTrials.gov).

Other drugs, both new and old, also have potential anti-adhesive effects that could be useful in SCD. P-selectin is known to contribute to adhesion of both sickle red cells and leukocytes to endothelial cells ^74– 76^, and heparins have both anti-P-selectin as well as anticoagulation effects ^75^. In fact, heparin blocks P-selectin-mediated adhesion at levels considerably lower than those needed for anticoagulation ^77^. A small phase 2 study of pentosan polysulfate sodium has shown promising results, in that a single oral dose improved microvascular blood flow, and repeated daily doses were associated with decreased plasma levels of soluble vascular cell adhesion molecule-1 (sVCAM-1) ^77^. Another compound chemically related to low-molecular-weight heparin (LMWH), sevuparin (Dilaforette), has shown promising results *in vitro* and *in vivo* in a mouse model of vaso-occlusion ^78^, and plans are under way to study the drug in SCD. Another P-selectin-targeted drug, SelG1 (Selexys Pharmaceuticals), is currently in clinical trial for use as a prophylactic agent to prevent vaso-occlusive crises (NCT01895361, ClinicalTrials.gov).

Finally, several studies in animals and patients have addressed the possibility that downregulation of signaling pathways may decrease cell adhesion. Several red cell adhesion receptors, including the BCAM/Lu receptor for laminin ^79^ and the ICAM-4 receptor for integrins ^80, 81^, are activated downstream of β-adrenergic receptor signaling pathways. Animal studies and a phase 1 trial of propranolol showed that propranolol decreased sickle red cell adhesion measured *in vitro* and decreased vaso-occlusion in mice *in vivo* ^81, 82^. In addition, the ERK signaling pathway appears to be involved in sickle red cell adhesion ^83, 84^, and the ability to affect this pathway via MEK inhibition is now being explored ^85^.

### Anticoagulants

Given the abundant data that coagulation pathways are abnormally activated in SCD, early studies explored the possibility that anticoagulation might have a beneficial effect in SCD, but most of those studies were too small or time-limited to be definitive. Using acenocoumarol, one study showed that achieving an international normalized ratio (INR) of 1.64 (range of 1.18–2.2) was associated with normalization of the F1 + 2 level and therefore concluded that low-intensity oral anticoagulation could normalize the hypercoagulability in SCD ^86^. Newer studies have again approached the potential usefulness of anticoagulation in SCD. A randomized double-blind clinical trial of an LMWH, tinzaparin, versus placebo was conducted during the management of acute painful vaso-occlusive episodes. This 253-patient study administered tinzaparin subcutaneously at 175 IU/kg once daily, along with usual supportive care and analgesia. Although the endpoints and criteria for discharge were different from those usually used in the United States and other Western countries, analysis demonstrated a statistically significant reduction in several measures of time to resolution ^87^. Another double-blind prospective study randomized SCD patients hospitalized for pain episodes to receive prophylactic LMWH (dalteparin 5,000 IU subcutaneously daily) or placebo. Although this study did not meet its target enrollment, the group receiving dalteparin had a greater decrease in pain scores at day 3 than did the placebo group (NCT01419977, ClinicalTrials.gov), although these results are unpublished to date. Another study used low-dose warfarin during vaso-occlusive crisis and studied D-dimer levels as their primary endpoint. They found that patients on warfarin had significantly lower D-dimer levels than patients not receiving the drug ^88^; however, effects on clinical endpoints, such as time to resolution of painful episode, were not reported. Finally, another study of acenocoumarol showed that treatment to INR values of 1.6 to 2 failed to lower the plasma levels of endothelial activation markers in SCD ^89^, raising questions about the likely clinical utility of anticoagulation to prevent SCD-related vascular events. Nevertheless, studies of the newer direct factor X inhibitors (apixaban and rivaroxaban) are currently planned or ongoing (NCT02179177 and NCT02072668, respectively, ClinicalTrials.gov). In addition, a study is under way to determine the feasibility of performing a larger multicenter phase III trial to assess the effects of unfractionated heparin in acute chest syndrome (NCT02098993, ClinicalTrials.gov).

Anti-platelet agents have also received attention and continue to be studied in the context of SCD. Eptifibatide is an anti-platelet agent that binds to the αIIbβIII integrin on platelets and decreased in platelet aggregation and sCD40L levels in patients with SCD in a phase I study ^90^. sCD40L is a pro-inflammatory cytokine released by platelets and chronically elevated in SCD plasma ^91^. However, use of eptifibatide in a small pilot study showed that eptifibatide did not improve the times to crisis resolution or hospital discharge ^92^. Another anti-platelet agent being studied in SCD is prasugrel. Recently, a multicenter phase 2 study of prasugrel versus placebo in adult patients with SCD showed that the drug could be safely used, and although it did not achieve statistically significant reductions in pain scores, it did reduce both platelet surface P-selectin and plasma soluble P-selectin levels, compared with placebo ^93^.

### Anti-inflammatory agents

Inflammatory pathways in SCD are both the result of red and white cell adhesion to endothelial cells and to each other as well as promoters of such adhesion. In addition, transient vaso-occlusion leads to hypoxia/reperfusion injury with a robust inflammatory component. Therefore, targeting inflammatory pathways is a rational approach to treating or trying to prevent vaso-occlusion in SCD. Corticosteroids have been investigated but with mixed results. Newer therapies are now addressing the role of invariant natural killer T (iNKT) cells, which are known to play an important role in ischemia/reperfusion injury and which are increased in both number and activity in SCD ^94^. In sickle mice, inhibition of iNKT cell activity with regadenoson, an adenosine A2A receptor agonist, led to a reduction in pulmonary inflammation and injury ^94^. A phase 1 study in patients also showed promising results, including reduction in phospho-NF-kappa-B p65 activation in iNKT cells, compared with pretreatment baseline during vaso-occlusion ^95^. The phase 2 study of regadenoson for treatment of vaso-occlusion is ongoing (NCT01788631, ClinicalTrials.gov). Another drug targeting iNKT cells is NKTT 120, a humanized monoclonal antibody against iNKT cells. After an initial study indicating safety, further ascending-dose phase 1 studies are being conducted to evaluate the safety, pharmacokinetics, pharmacodynamics, and biologic activity of the drug (NCT01783691, ClinicalTrials.gov).

### Other therapeutic approaches

Although the salutary effect of HU on SCD may be due to a more complex mechanism than its ability to raise HbF levels in about 50% of patients, it is also clear that not everyone responds to HU either clinically or with appreciably higher HbF levels. Therefore, other ways to increase HbF continue to be explored. Early studies showed that decitabine could substantially increase HbF levels in HU-non-responders ^96^ and that such increased HbF levels were sustainable with repeated intermittent treatment ^97^. Decitabine continues to be studied, both alone (NCT01375608, ClinicalTrials.gov) and in combination with tetrahydrouridine, a competitive inhibitor of cytidine deaminase that is being studied in an effort to improve oral bioavailability of decitabine ^98^ (NCT01685515, ClinicalTrials.gov). Other agents that are being studied for their effects on HbF levels include panobinostat, vorinostat, pomalidomide, arginine butyrate, and HQK-1001 (2,2-dimethylbutyrate) ^99– 101^. However, in a phase 2 study, HQK-1001 was associated with only a modest HbF response and a paradoxical increase in vaso-occlusive episodes ^101^.

## Implications for the future

The complexity of the pathogenesis of SCD has resulted in a plethora of potential druggable targets in our effort to ameliorate the disease’s sequelae. However, this same complexity has also prevented us from knowing which targets are optimal ones. Biomarkers may be helpful in patient selection for both research studies or therapy, but proof that changes in biomarkers are associated with clinical improvement remains elusive in most instances. Therefore, until we achieve wide availability of curative therapies through either hematopoietic stem cell transplantation or gene therapy, we need to continue our search for therapies that can be provided either to prevent or treat acute disease manifestations, such as vaso-occlusive pain, and chronic organ damage, such as sickle cell nephropathy and pulmonary hypertension. These goals, however, are still not quite within reach.
